# Time to put a spotlight on out-patient chronotherapy for depression

**DOI:** 10.1192/bjo.2021.1056

**Published:** 2021-11-24

**Authors:** Havard Kallestad, Jan Scott

**Affiliations:** Division of Mental Health Care, St Olavs University Hospital, Trondheim, Norway; and Division of Mental Health Care, Department of Research and Development, Norwegian University of Science and Technology, Trondheim, Norway; Department of Academic Psychiatry, Newcastle University, UK; and Division of Mental Health Care, Department of Research and Development, Norwegian University of Science and Technology, Trondheim, Norway

**Keywords:** Sleep deprivation, chronotherapy, depression, master clock, out-patient treatment

## Abstract

The challenge of identifying efficacious out-patient treatments for depression is amplified by the increasing desire to find interventions that reduce the time to sustained improvement. One potential but underexplored option is triple chronotherapy (TCT). To date, use of TCT has been largely restricted to specialist units or in-patients. Recent research demonstrates that it may be possible to undertake sleep deprivation in out-patient settings, raising the possibility of delivering TCT to broader populations of individuals with depression. Emerging evidence suggests that out-patient TCT is a high-benefit, low-risk intervention but questions remain about how to target TCT and its mechanisms of action. Like traditional antidepressants, TCT probably acts through several pathways, especially the synchronisation of the ‘master clock’. Availability of reliable and valid methods of out-patient measurement of intra-individual circadian rhythmicity and light exposure are rate-limiting steps in the wider dissemination of TCT.

In 2017, depression was a greater contributor to the global burden of disease than any other mental disorder, accounting for 43 out of 142 million disability-adjusted life-years associated with these conditions.^1^ After more than 1000 randomised controlled trials (RCTs) of physical, pharmacological and psychological treatments, identifying optimal antidepressant interventions remains a challenge.^2^ Two contemporary concerns are the need to better understand who will engage with and benefit from different treatment options^2^ and to identify interventions that reduce the time to sustained improvement.^3^ Several recent RCTs have highlighted the potential for a rapid onset of antidepressant effects of ketamine, although gains may be lost within days or weeks.^4^ A similar response pattern was previously observed in studies of sleep deprivation, with dramatic reductions in depression after 24–48 h of prolonged wakefulness, but early symptomatic relapse the morning after restoration of normal sleep (so-called recovery sleep).^5^ However, further experimentation identified that the combination of sleep deprivation with sleep-phase advance and light therapy (so-called triple chronotherapy, TCT) could lead to rapid and sustained response in major depression.^5,6^ To date, the applications of TCT have been largely restricted to seasonal affective disorders, mood disorders treated in tertiary or specialist units, or to in-patients.^5,6^. There is a previous RCT of the intervention for medication-treated out-patients with bipolar disorders from the USA^7^ and a more recent a Dutch study of its feasibility and acceptability in 27 medication-free out-patients with major depression (about 20 participants also received cognitive–behavioural therapy at some point).^8^ Out-patient TCT appeared to have a high benefit/risk ratio in bipolar depression and produced early and sustained improvement for participants with unipolar depression.^7,8^ Consequently, the UK-based trial published by Veale and colleagues in this issue of *BJPsych Open* is important and timely.^3^

## The Veale study's findings

In their pilot RCT of 82 out-patients with depression, Veale at al found significant benefits from TCT compared with the control intervention.^3^ For example, from the response rates reported in intent-to-treat (ITT) analyses, we estimate that the number needed to treat (NNT) is 6 (95% CI 2.7–75.7) at 2 weeks and 5 by about 6 months follow-up (95% CI 2.5–27.2). These estimates and other reported outcomes suggest that, compared with the control intervention, TCT is associated with a rapid and sustained antidepressant response of substantial magnitude. However, besides the very wide confidence intervals for these NNT (which are inevitable in a small pilot trial), there are other reasons to proceed with caution. Sample attrition rates were high, and of 173 individuals with major depression who were originally screened, only 47 (27%) completed the RCT. It was noticeable that drop-out both before and after commencing the trial was higher among those allocated to TCT. Given that out-patient TCT appears to be a low-risk intervention, it will be important to review how the rationale is presented and clarify whether there is any real-world reluctance to commence this intervention (and resolve any authentic concerns). Also, if TCT is to be translated into day-to-day clinical settings, then there are some practical issues to address, particularly the availability of sleep deprivation sessions and staff trained to deliver this intervention. Notably, some individuals had to wait 3–4 weeks to undertake this first rate-limiting step of the TCT programme. In reality, this delay means that the time between giving consent to receive TCT and experiencing a meaningful response could equal the latency between starting an antidepressant medication and showing clinical improvement (i.e. 4–6 weeks). Although the availability of sleep deprivation sessions might differ outside the confines of an RCT, increasing the frequency of these sessions has resource implications that will have an impact on the cost-effectiveness of TCT. A possible solution might be to explore the feasibility of out-patients undertaking sleep deprivation independently in their own home, but adherence and efficacy may be undermined if such less stringent alternatives are employed. On a similar theme, the relative importance of the adjunctive use of blue-blocking glasses (BBG) and the duration of morning bright-light therapy will need to be reviewed in future comparative-effectiveness RCTs. This is required first because recent reviews question the added value of BBG for depression^9^ and second because we lack evidence that 6 months of morning bright-light therapy is an optimal or achievable regime (in terms of daily adherence).^10^ For example, the RCT in bipolar depression achieved good outcomes with a much shorter duration of light therapy.^7^ As the up-front purchase of the equipment modifies the overall cost–benefit estimates (especially if light boxes were provided for the sole use of each out-patient for 6 months and rates of return of BBG and light boxes will probably be 50–60%), it is important to determine the required dose and duration of these elements more precisely.

## Conclusions

Despite acknowledged limitations associated with the pilot trial, it is still a very useful contribution to the literature and helps point to the next step in out-patient TCT research.^3^ Critically, it will be important to consider which individuals with depression should be targeted for this intervention. None of the existing out-patient studies of TCT were able to explore moderators or mediators,^3,7,8^ although there is no evidence that concurrent receipt of antidepressant medication and/or therapy either enhanced or undermined rapid response to TCT. In Veale et al's RCT, the response rates in the control group were low (even though many were receiving therapy and/or medication) and so future studies will need to consider the impact on outcome of the recruited case mix (i.e. proportions of dysthymia and chronic depression, and unipolar and bipolar depressions). Similarly, Veale et al evaluated sleep patterns using the self-rated Pittsburgh Sleep Quality Index (PSQI). Few between-group differences in PSQI scores emerged, which might reflect the problem that PSQI self-ratings can differ significantly from objective recordings (such as actigraphy).^11^ Alternatively, it may be because PSQI scores measure sleep quality and quantity rather than circadian rhythmicity. Identifying the most appropriate means of monitoring the timing and synchronisation of sleep–wake cycles in TCT studies will be essential. Fine-tuning of the TCT intervention (e.g. required phase shifts) relies on such estimates to some extent.^3,5,12^ Equally, these investigations may help shed light on the mechanisms of action of TCT. It is postulated that chronotherapies affect mood directly but also indirectly via their effects on sleep and the synchronisation of the timing of the internal master pacemaker (or ‘biological clock’) and stabilisation of circadian rhythms.^12,13^ However, evidence is largely circumstantial that chronotherapies actually manipulate the biological clock itself and a rate-limiting step in clarifying the mechanisms of action of TCT is the difficulty in identifying valid direct methods of assessment that can be used in day-to-day practice reliably. There are several potential tools and tests that should be transferable from bench to bedside that could provide an objective and contact-free estimate of sleep–wake phase in the near future.^14–17^ These developments may provide the impetus needed for both research and clinical practice as they could be used for assessing whether someone with depression may benefit from TCT or not and could be used for better accuracy and individual adaptation when determining the timing of some chronotherapeutic methods such as light and darkness exposures.^14–17^ Then perhaps we will see more widespread application of these undervalued yet potentially useful interventions.

## Data Availability

Data availability is not applicable to this article as no new data were created or analysed in its preparation.
